# Does a surgical mask improve oxygenation in COVID-19 patients?

**DOI:** 10.1186/s40981-021-00439-7

**Published:** 2021-04-14

**Authors:** Yusuke Matsui, Tomonori Takazawa, Akihito Takemae, Shigeru Saito

**Affiliations:** Intensive Care Unit, Gunma University Hospital, 3-39-15 Showa-machi, Maebashi, Gunma 371-8511 Japan

To the Editor,

For anesthesiologists and operating room nurses, protection from coronavirus disease 2019 (COVID-19) infection while on duty is currently a top priority. According to the recommendation issued by the Japanese Society of Anesthesiology in March 2020, following extubation at the end of general anesthesia, the patient should receive oxygen via an oxygen mask placed over a surgical mask [1]. The rationale for this recommendation is the inhibitory effect of the surgical mask on the spread of respiratory aerosols. Binks and colleagues recently investigated whether the fraction of inspired oxygen (FiO_2_) was different when wearing surgical masks above and below the oxygen mask. They suggested that oxygen supply via the oxygen mask was not disturbed by simultaneous use of a surgical mask [2]. From a slightly different point of view, however, COVID-19 patients with severe hypoxia might require oxygen administration for a long time after extubation. We devised a method of delivering oxygen through a nasal cannula under a surgical mask to obtain maximum oxygenation while preventing aerosol dispersal (Fig. 1). We compared our method with the method recommended by Binks et al. in a patient with COVID-19 being treated at our intensive care unit.

**Fig. 1:**
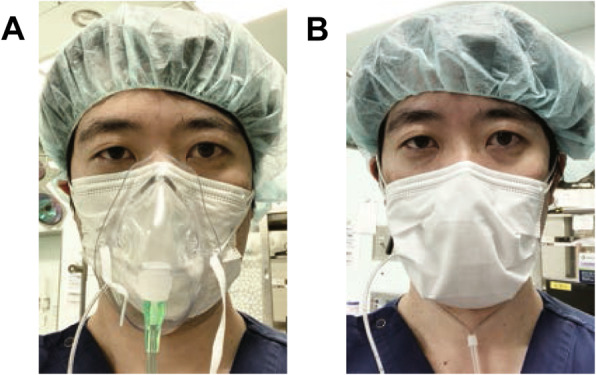
The two different oxygen administration methods tested. Image showing wearing an oxygen mask above the surgical mask (**a**) and wearing a nasal cannula below the surgical mask (**b**)

The results are shown in Table 1. Partial pressure of oxygen in arterial blood (PaO_2_) clearly decreased when changing from the nasal cannula to the oxygen mask, recovering once again when returning to the nasal cannula. In contrast, other indices, such as pH, partial pressure of carbon dioxide in arterial blood (PaCO_2_), base excess, and respiratory rate, remained almost unchanged. These results suggest that oxygen delivered via a nasal cannula worn under a surgical mask might prevent the spread of infection while simultaneously allowing maintenance of a high PaO_2_ in patients.

**Table 1:** Results of blood gas analysis in the COVID-19 patient

Oxygen supply device	Nasal cannula	Oxygen mask	Nasal cannula
Oxygen flow rate (L/min)	4	4	4
pH	7.38	7.41	7.41
PaO_2_ (mmHg)	154	108	150
PaCO_2_ (mmHg)	36.1	32.9	32.6
Base excess (mmol/L)	−3.5	−3.4	−3.8
Respiratory rate (breaths/min)	29	30	28

We collected arterial blood samples and performed blood gas analysis when the patient wore a nasal cannula under the surgical mask and when the patient wore an oxygen mask over the surgical mask. Blood samples were taken between 15 and 60 min after the change in oxygen administration method, i.e., after the patient received oxygen for at least 15 min by the method being tested. This was to clarify that the results of blood gas analysis were due to the different methods of oxygen administration. Arterial blood was analyzed by a blood gas analyzer (ABL800 FLEX, Radiometer Medical ApS, Denmark). *PaO*_*2*_, partial pressure of oxygen in arterial blood; *PaCO*_*2*_, partial pressure of carbon dioxide in arterial blood

To verify why our method was advantageous for oxygenation, we decided to estimate inhaled oxygen concentration when oxygen was administered to a healthy subject. Due to technical difficulties, we compared oxygen levels in exhaled air rather than inhaled air: Exhaled oxygen concentration was 23% with the oxygen mask, and 33% with the nasal cannula (Supplemental Figure 1). Considering that oxygen consumption during these measurements was constant, the difference in exhaled oxygen concentration can be interpreted as a reflection of the difference in inhaled oxygen concentration.

A limitation of our report is that we do not know why our method resulted in an enhanced FiO_2_, leading to the higher PaO_2_. Although it is controversial whether the oxygen mask or nasal cannula is more advantageous for oxygenation [3, 4], wearing a surgical mask over the cannula improved oxygenation in COVID-19 patients in a study with high-flow nasal cannula treatment [5]. Details about the mechanism for the superiority of our method are unknown and need to be clarified.

We were able to obtain good oxygenation in a patient with COVID-19 by supplying oxygen via a nasal cannula used below a surgical mask. We need to investigate this method in other COVID-19 patients in the future. Additionally, this method could be applied to patients other than those with COVID-19.

## Supplementary Information

**Additional file 1: Supplemental Figure 1.** Results of gas analysis in a healthy male volunteer. The figures show the results of gas analysis when 4 L/min of oxygen was administered via an oxygen mask worn over a surgical mask (A) and via a nasal cannula worn below a surgical mask (B). The subject was instructed to hold the tip of the gas sampling line in his mouth. He was asked to breathe at a respiratory rate of about 15-17 breaths/min, and when his EtCO_2 _stabilized at about 40 mmHg, the oxygen concentration in exhaled air was assessed and compared between the two methods. A multi-gas analysis unit (GF-320R, Nihon Kohden Corporation, Tokyo) was used for the measurement. RR, respiratory rate; O_2_-E, oxygen concentration in exhaled air; O_2_-I, oxygen concentration in inhaled air; CO_2_-E, carbon dioxide concentration in exhaled air; CO_2_-I, carbon dioxide concentration in inhaled air.

## Data Availability

Data relevant to this letter are not available for public access because of patient privacy concerns, but are available from the corresponding author on reasonable request.

## Abbreviations

FiO_2_: Fraction of inspired oxygen
COVID-19: Coronavirus disease 2019
PaO_2_: Partial pressure of oxygen in arterial blood
PaCO_2_: Partial pressure of carbon dioxide in arterial blood

## References

[1] Japanese Society of Anesthesiologists (2020). Our response to coronavirus disease 2019 (COVID-19).

[2] Binks AC, Parkinson SM, Sabbouh V (2020). Oxygen: under or over a surgical facemask for COVID-19 patients?. Anaesthesia..

[3] Ayhan H, Iyigun E, Tastan S, Orhan ME, Ozturk E (2009). Comparison of two different oxygen delivery methods in the early postoperative period: randomized trial. J Adv Nurs..

[4] Stausholm K, Rosenberg-Adamsen S, Skriver M, Kehlet H, Rosenberg J (1995). Comparison of three devices for oxygen administration in the late postoperative period. Br J Anaesth..

[5] Montiel V, Robert A, Robert A, Nabaoui A, Marie T, Mestre NM, et al. Surgical mask on top of high-flow nasal cannula improves oxygenation in critically ill COVID-19 patients with hypoxemic respiratory failure. Ann Intensive Care. 2020;10(1):125. 10.1186/s13613-020-00744-x. 10.1186/s13613-020-00744-x PMC7523252 32990864

