# Supplementary figures and images for: Does a surgical mask improve oxygenation in COVID-19 patients?
Source: JA Clin Rep. 2021 Apr 14;7:34. doi: 10.1186/s40981-021-00439-7 (PMC8045437; doi:10.1186/s40981-021-00439-7)

A

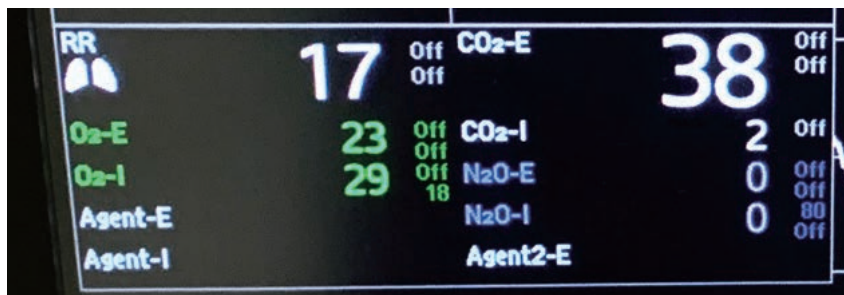

B

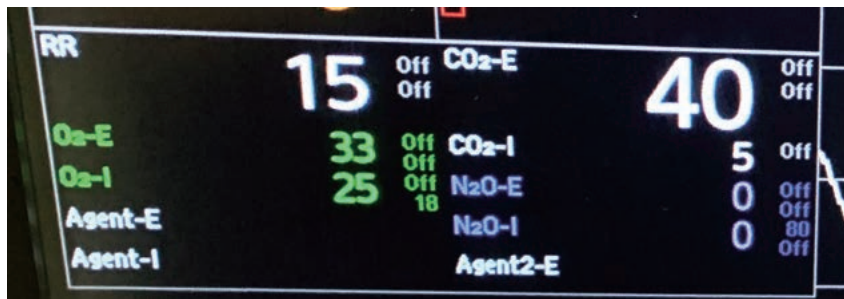

Supplement: Supplementary file 1 — Additional file 1: Supplemental Figure 1. Results of gas analysis in a healthy male volunteer. The figures show the results of gas analysis when 4 L/min of oxygen was administered via an oxygen mask worn over a surgical mask (A) and via a nasal cannula worn below a surgical mask (B). The subject was instructed to hold the tip of the gas sampling line in his mouth. He was asked to breathe at a respiratory rate of about 15-17 breaths/min, and when his EtCO2 stabilized at about 40 mmHg, the oxygen concentration in exhaled air was assessed and compared between the two methods. A multi-gas analysis unit (GF-320R, Nihon Kohden Corporation, Tokyo) was used for the measurement. RR, respiratory rate; O2-E, oxygen concentration in exhaled air; O2-I, oxygen concentration in inhaled air; CO2-E, carbon dioxide concentration in exhaled air; CO2-I, carbon dioxide concentration in inhaled air. [file 40981_2021_439_MOESM1_ESM.pdf]
